# Supplementary material for: Protocol for designing and testing the effectiveness of a post caesarean section home care guide in preventing surgical site infection in Central Tanzania
Source: PLoS One. 2023 Jun 14;18(6):e0287004. doi: 10.1371/journal.pone.0287004 (PMC10266676; doi:10.1371/journal.pone.0287004)
Supplement: Attachment — Submitted filename: Response to reviewers.docx [file pone.0287004.s001.docx]

**Responses to comments made by Reviewers:**

Authors thank the reviewers for the constructive comments that in our view we believe will improve our work to meet the quality standards of this prestigious journal. The table below presents our responses to the comments.

| **S/N** | **Comments** | **Response** | **Page Number** |
| --- | --- | --- | --- |
| 1. | Please ensure that your manuscript meets PLOS ONE's style requirements, including those for file naming. The PLOS ONE style templates can be found at  https://journals.plos.org/plosone/s/file?id=wjVg/PLOSOne_formatting_sample_main_body.pdf and  https://journals.plos.org/plosone/s/file?id=ba62/PLOSOne_formatting_sample_title_authors_affiliations.pdf | The manuscript has been edited to meet journal requirement | Entire document |
| 2. | We note that the grant information you provided in the ‘Funding Information’ and ‘Financial Disclosure’ sections do not match. When you resubmit, please ensure that you provide the correct grant numbers for the awards you received for your study in the ‘Funding Information’ section. | The award received for the PhD program not even for the protocol development. The funds used were for the faculty development | NA |
| 3. | Thank you for stating the following in the Acknowledgments Section of your manuscript: "On behalf of all authors we acknowledge the redness of University of Dodoma for funding the study."  We note that you have provided funding information that is not currently declared in your Funding Statement. However, funding information should not appear in the Acknowledgments section or other areas of your manuscript. We will only publish funding information present in the Funding Statement section of the online submission form.  Please remove any funding-related text from the manuscript and let us know how you would like to update your Funding Statement | The University of Dodoma was acknowledged by funding the PhD study specifically for paying tuition fees and research study. This information is removed in this section and will be included in its appropriate section in the online submission form. | NA |
| 4. | In your revised cover letter, please address the following prompts:   a) If there are ethical or legal restrictions on sharing a de-identified data set, please explain them in detail (e.g., data contain potentially sensitive information, data are owned by a third-party organization, etc.) and who has imposed them (e.g., an ethics committee). Please also provide contact information for a data access committee, ethics committee, or other institutional body to which data requests may be sent. b) If there are no restrictions, please upload the minimal anonymized data set necessary to replicate your study findings as either Supporting Information files or to a stable, public repository and provide us with the relevant URLs, DOIs, or accession numbers. For a list of acceptable repositories, please see <http://journals.plos.org/plosone/s/data-availability#loc-recommended-repositories>.   We will update your Data Availability statement on your behalf to reflect the information you provide. | According to our policy, the data from this study are owned by the Muhimbili University of Health and Allied Science- Research Ethics Committee (MUHAS-REC). However, they may be requested from the Directorate of Research and Publication. Email: [drp@muhas.ac.tz](mailto:drp@muhas.ac.tz)  This information is included in the cover letter as advised | NIL |
| 5. | Please also have someone edit this to ensure correct English grammar. Standard proofreading (fixing small errors related to tense and number) would eliminate many errors. | The manuscript has been English edited for clarity as advised. | Entire document |
| 6. | Please include captions for your Supporting Information files at the end of your manuscript, and update any in-text citations to match accordingly. Please see our Supporting Information guidelines for more information: http://journals.plos.org/plosone/s/supporting-information. | The caption and the citation of the files have been properly corrected according to Plos One guideline | 8,9,27,33 |
| 7. | Thank you for the opportunity to review the paper titled “Designing and testing the effectiveness of a post caesarean section home care guide in preventing postpartum complications in Central Tanzania; Protocol.” I am very enthusiastic about the proposed research as I agree that there is a dearth of research on this topic and currently poor practice in this area.  Unfortunately, there are numerous errors in writing throughout the manuscript. Below, I highlight some of these issues on the first few pages, but as the authors will note, there are so many that this was not possible to do for the entire paper. I strongly recommend engaging in a peer-editor to clean this up so that the very valuable content is not lost in minor errors in writing.  Even with concerns in the writing, the content is clear enough for my enthusiasm to remain. I do highlight a few technical points for them to consider in this next stage | The manuscript has English edited for clarity by the professional English editor. | Entire documents |
| 8. | Reference 2 is dated. | The reference has been corrected | 26 |
| 9. | More facility delivery is the only reason there is more c/section (last line first paragraph, page 4), and so that needs to be expanded. Then when I looked into the reference to understand what that paper is saying, reference 3 is for “Five national academies call for global compact on air pollution…” This is clearly not the right reference.  To that end – please check *ALL* for your references. Many of them are incorrectly formatted.  In addition, I want to call your attention to a few new articles recently out that should be included. None of these negate this proposal. If anything, it supports the claim that this area is critically neglected. - Discharge instructions given to women following delivery by cesarean section in Sub-Saharan Africa: A scoping review. Musabeyezu et al. https://doi.org/10.1371/journal.pgph.0000318 - New global WHO postnatal guidance is welcome but misses the long-term perspective. Bick et al. - Also look up the WHO guidelines referenced in the Bick et al paper. | All references have been cross checked for correctness and completeness and formatted accordingly.  One of the proposed article was already considered, the rest of the proposed studies have been read and included in the documents as well to support the study problem. | 5, 6, 31  6,7 |
| 10. | The search criteria need a lot of work. Some examples: - If I just copy and paste the first bullet into pubmed, nothing comes up. That is concerning. - There are many ways to spell cesarean; your terms only look at one. - By looking at “post cesarean section” together in quotes, then you miss just cesarean. I write a lot about post-c-section surgical site infections. In none do I write “post cesarean section” exactly. | The suggestion has been taken with great consideration, search words have been updated to cover broad range of literature. | 11,12 |
| 11. | You need more detail than “hand searching” | Searching words of guidelines from Ministry of health were also be added | 11 |
| 12. | Will you use multiple coders in your qualitative analysis? | Yes, we will use multiple coders, and agreed on the final codes and themes | N/A |
| 13. | Be careful – it is not a rickets scale | We acknowledge it is a typing error. We have corrected it to read “Likert scale ˮ | 16 |
| 14. | Can you provide the tool described on Page 16 for knowledge assessments? | To the best of our knowledge, there is no tool to assess knowledge on home care after caesarean section; it is a primary objective of the current study to develop home care guide. From this, training and tool for knowledge assessment on home care after CS will be developed and tested accordingly. | N/A |
| 15. | For your sample size calculation, Your z_beta is for 80% power; for 90% power, you need 1.282. Fine to just clarify and power at 80% power. | Thank you for the important observation, the correction has been made accordingly. | 19,20 |
| 16. | For your sample size calculation – not totally unreasonable, but not conventional to put at exact observed levels from other studies. It is fine to keep, but also fine to let those studies guide you but not completely drive what you do. | To the best of our understanding, findings from other studies were placed to help estimate the sample size in this study. However; in the formula there is a mean while the adopted levels are in proportion, the formula has been changed (from mean to proportion) to reflect the proportion in the adopted study. | 19,20 |
| 17. | I really don’t expect that your intervention will cut the SSI rate by 2/3. (From 16% to 4.9%). Personally, I would not power for this as: 1) Even if a good intervention, this is not the sole (or even primary) driver of SSI. 2) Even if you don’t have significant declines in SSI rates, good messaging/communication to moms has lots of benefits. | The adopted study cut off the SSI on the previous study from 16% to 4.9; that was used to estimate the sample size in this study. We also don’t expect the same as the output of the study will depend on the quality of intervention, and many more factors. We are glad that the study will send and important study to the nurse midwives and mothers. | NIL |
| 18. | You power for binary measures, but then your primary analysis (page 24) treats scores as continuous. Either need to change your sample size calculation approach or your analysis approach. | This study has both continuous (knowledge of study participants on home care after caesarean section) and categorical variables (Surgical site infection-SSIs). We have added the description of the analysis of the continuous data clearly. | 24,25 |
| 19. | Editing issues found on the first few pages. (As noted above, it was not possible to make this level of editing throughout, but this is hopefully illustrative of what will need to be done throughout.) • Page 2, second line. Should be plural “infections”. Also needs to be SSIs. Need to check throughout the paper, as anywhere there is SSI it may, or may not, need to be written as SSIs. • Page 2, make validation plural: “Following a series of validations…” • Page 4, first line, need a comma between intervention and performed. “…. Intervention, performed to…” • Page 4, change from into to in, to read “… may result in complications…” • Page 4, “…hospitalization, increase health cost,…” add ‘d’ to increase, fix spelling, add s to cost, to read “… hospitalization, increased health costs,…” • Page 4, add “and” to “… for her baby, and maternal death.” | Thank you for the important observation, I admit there were typing errors and all have been corrected. | Entire document |
| **Reviewer 2** | | |  |
| 1. 29. | This study has a nice design--the mixed methods approach is appropriate. Please clarify "intervention" on p. 8: "...evaluation of the intervention in increasing knowledge of home care and preventing postpartum complications." Are you evaluating the training or the guide? How would you separate training and guide in this circumstance. | The primary goal of the study is to design and test the effectiveness of the home care GUIDE in preventing SSI. So we evaluate the effectiveness of the developed guide, in it we will also assess participant’s (nurse midwives and post CS mothers) opinion, on the intervention used to deliver the home care education. Hence at the end we will be able to recommend the guide and the suitable intervention approach that might be used to deliver the home care knowledge basing on the proposed opinion of participants. | NA |
| 2. | I don't understand this sentence: "Data extraction will be guided by the validated to ensure collection of relevant data from all retrieved document, risk of errors in transcription is minimized, to guarantee precision in data collection and to serve as a record (30)." | The sentence has typing error; it has been edited for easy understanding.  It meant that validated tool will be used to data regarding home care practice after caesarean section from retrieved documents. By so doing it will help to correct uniform and relevant information, and also it will help to save as a record of information’s gathered from the retrieved documents. | 10,11 |
| 3. | Purposive sampling technique (32) will be used to recruit 5-25 participant (Creswell, 2016) based on principle of data saturation (33)." Which participants? midwives or mothers? Do you intend to conduct interviews until you've reached saturation? You could consider making full transcriptions of interviews for accuracy and easier coding. For the post-test with mothers, will you also evaluate if the recommended practices were applied? I don't think 30 mothers are enough to use multivariable regression. Your question for multivariable analysis is not clear. What is the exposure? All mothers were counselled on the new guidance. (Phase 3). | In qualitative phase, we meant to use purposive sampling will be used to recruit both nurse midwives and post caesarean section mothers. And to all interviews will be conducted.  Yes, full transcription of in-depth interviews will be done before coding. The statement has been added in the revised documents to make it clear.  In pre-testing the guide and tool, we changed the number to each type of participant, we have use 20% of the obtained sample size for them to be exposed with the guide and tool. The number has then increased from 30 estimated sample size to 50 nurse midwives and 83 post CS mothers; enough to use multivariable regression. The information has been added in the documents | N/A  13  15 |
| 4. | On p. 17 you introduce prevention of post-partum anemia. But the purpose of the guide to that point is to prevent SSI. Preventing, identifying, treating anemia involves a different protocol. Many factors contribute to anemia | We have decided to exclude anaemia issue; and better concentrating with SSI and knowledge of home care after caesarean section only. The concept of anaemia has been removed from the document. | Entire documents |
| 5. | Why not randomize mothers to the intervention? How will you choose controls (the non-equivalent control group)? Will you match on certain characteristics? That decision has implications for the analysis. How will you prevent "contamination" in the education and training of midwives to those not involved with the intervention? | According to our understanding; the basic of quasi experimental studies lacks randomization. Hence Dodoma Regional Referral Hospital will be and interventional study and Singida referral Regional Hospital will be the control. This has been decided to reduce the risk of contamination of intervention as it is hard to tell a nurse midwife that ‘you have to educate this woman but not this’.  The ratio of interventional to control group will be 1 to 2 whereby there will be matching between the group in terms of maternal age, gravidity, type CS; this information has been added in the document. | N/A  19 |
| 6. | The mothers cared for by the intervention midwives will be the intervention mothers. For controls, you will have to enrol 2 mothers cared for by non-intervention midwives, correct? Text on p.23 indicates the intervention assignment is by hospital; I do not see that explained earlier in the protocol. | Yes; for controls, post CS mothers will be cared by non-interventional mother at Singida Regional Referral hospital while for interventional post CS mothers will be cared by interventional nurse midwives at Dodoma regional referral hospital. The explanation has been clearly added in the documents | 21 |
| 7. | To mitigate other causes of SSI, you might create a scoring system based on info in patients' files that indicates risk of SSI from issues other than home care. You could then control for that score. | Thank you for a very constructive comment, we will develop the tool basing on the existing studies and other literature. | N/A |
| 8. | The dependent variables you list in the figure--shouldn't they be just knowledge and SSI? Are you following mothers to determine if SSI develops? For how long will you follow them? State the dependent and exposure variables clearly for each quantitative assessment. | Yes, those are the dependent variables, they have been allocated clearly in the text.  I am following mothers to determine SSI in three post-natal visits (7^th^, 14^th^ and 30^th^ day) after operation. | 24 |

Kindly receive the responses of the authors to the raised comments, we would like to clarify more in case of unsatisfactory response; aiming at improving our work.

Thank you very much

On behalf of all authors,
