# Supplementary material for: Protocol for designing and testing the effectiveness of a post caesarean section home care guide in preventing surgical site infection in Central Tanzania
Source: PLoS One. 2023 Jun 14;18(6):e0287004. doi: 10.1371/journal.pone.0287004 (PMC10266676; doi:10.1371/journal.pone.0287004)
Supplement: Attachment — Submitted filename: rebuttal letter phase 5.docx [file pone.0287004.s003.docx]

On behalf of all other, we are thankful for receiving the constructive comments from the reviewers.

Below is the table of comments and their responses.

| **S/N** | **COMMENTS** | **RESPONSE** | **PAGE** |
| --- | --- | --- | --- |
| 1. | Please review your reference list to ensure that it is complete and correct. If you have cited papers that have been retracted, please include the rationale for doing so in the manuscript text, or remove these references and replace them with relevant current references. Any changes to the reference list should be mentioned in the rebuttal letter that accompanies your revised manuscript. If you need to cite a retracted article, indicate the article’s retracted status in the References list and also include a citation and full reference for the retraction notice. | 1. The references have been edited; two references have been removed because are retracted references (number 10 and 30). 2. Most have been improved and the link or ‘doi’ number have been added, in Mendeley if you delete the in text citation the reference is automatically deleted hence it is not seen as strikethrough 3. | 31-37 |
| 2. | You use the terms "mother", "woman", and "female" throughout the manuscript. Please use one term consistently. I suggest using "woman." | The term woman has been used throughout the manuscript | Entire document |
| 3. | Please again review for English grammar word usage and punctuation. It gets better with each revision but there are still approximately two errors on each page.  For example, there are several instances where you use a semi-colon incorrectly, as in the Abstract methodology section. The journal does not provide editing services so you want your submitted manuscript to be correct. | The English grammar, word usage and punctuation have been revised in the entire document | Entire Document |
| 4. | There are 5 descriptions of search terms but only four questions (p.10). It's not clear why you would conduct 5 separate searches. | The fifth question was missing; it has been added.  Regarding five separate search; this is because if you search home care after caesarean delivery you will find not results until you separate the care is when you will find articles. I tried that, after failing I got an advices to search items separately | 9 |
| 5. | P. 11--"data extraction will be guided by the validated tool..." What is the tool you are referring to here? | The tool has been adopted from Elizabeth Ursi and Cristina Gavao as cited by Marcela Souza. The citation has been added in the documents and attachment of the tool has been added in the submission site | 11 |
| 6. | P. 29 study power. You say 80% but then have (0.84). Is that a typo? | Yes it is a typo, and it has been corrected from 0.84 to 0.80 | 21,22,28 |
| 7. | Do you want to add a Limitations section to the Discussion? If so, please include the possibility of uncontrolled confounding in the estimates derived from the statistical models. | The limitation section have been added | 30 |

The Validated tool used to collect the integrative review data

**Data collection instrument (By Ursi, 2005).**

| **A. Identification** |  |
| --- | --- |
| Title of the article |  |
| Title of the journal |  |
| Authors |  |
| Country |  |
| Language |  |
| Year of publication |  |
| **B. Institution holding the study** |  |
| Hospital |  |
| University |  |
| Research center |  |
| sole institution |  |
| Multicenter study |  |
| Other institutions |  |
| No identification of setting |  |
| **C. Type of publication** |  |
| Nursing publication |  |
| Medical publication |  |
| Publication in another area of health. Which area? |  |
| **D. Methodological characteristics of the study** |  |
| 1. Type of publication |  |
| 2. Objective or investigation question |  |
| 3. Sample |  |
| 4. Treatment of Data |  |
| 5. Intervention performed |  |
| 6. Results |  |
| 7. Analysis |  |
| 8. Implication |  |
| 9. Evidence level |  |
| **E. Assessing methodological rigor** | |
| Clarity to identify the methodological steps in the text (method employed, participating subject, inclusion/ exclusion criteria, intervention, results) |  |
| Identification of restriction of Bias |  |
