# Supplementary material for: Protocol for designing and testing the effectiveness of a post caesarean section home care guide in preventing surgical site infection in Central Tanzania
Source: PLoS One. 2023 Jun 14;18(6):e0287004. doi: 10.1371/journal.pone.0287004 (PMC10266676; doi:10.1371/journal.pone.0287004)
Supplement: Attachment — Submitted filename: rebuttal letter phase 7.docx [file pone.0287004.s005.docx]

On behalf of all other, we are thankful for receiving editorial constructive comments.

Below is the table of responses from authors

| **S/N** | **COMMENTS** | **RESPONSE** | **PAGE** |
| --- | --- | --- | --- |
| 1. | Please ensure that you refer to Figure 1 & 2 in your text as, if accepted, production will need this reference to link the reader to the figure. | Thank you for the comments, citation of S1_Fig has been removed as instructed previously. We have replaced them by referring to fig 1, 2 and 3 | 6,8 and 26 |
| 2. | Please upload a new copy of Figure 1 & 2 as the detail is not clear. Please follow the link for more information: <https://blogs.plos.org/plos/2019/06/looking-good-tips-for-creating-your-plos-figures-graphics/> | The figure has been uploaded and the new version has been submitted in the system | N/A |
| 3. | Please include a separate legend for each figure in your manuscript. | Separate legends for each figure have been added to the manuscript. Figure captions have also been added in the manuscript as per authors instructions of the journal | 6,8,26,35-37 |
| 4. | At this time, please address the following queries:  a)        Please clarify the sources of funding (financial or material support) for your study. List the grants or organizations that supported your study, including funding received from your institution.  b)        State what role the funders took in the study. If the funders had no role in your study, please state: “The funders had no role in study design, data collection and analysis, decision to publish, or preparation of the manuscript.”  c)        If any authors received a salary from any of your funders, please state which authors and which funders.  d)        If you did not receive any funding for this study, please state: “The authors received no specific funding for this work.”  Please include your amended statements within your cover letter; we will change the online submission form on your behalf. | - The source of fund or this study is the University of Dodoma, that covered both the tuition fee for the PhD and the data collection costs. - The funders had no role in study design, data collection and analysis, decision to publish, or preparation of the manuscript. - Mdoe Mwajuma B. and Kibusi, Stephen M. are the employees of the University of Dodoma and they receive their salary from this institution. | N/A |
